# Energy balance and cancer risk at the cellular and whole organism level: modification by metformin

**DOI:** 10.1186/1753-6561-6-S3-O21

**Published:** 2012-06-01

**Authors:** Michael Pollak

**Affiliations:** 1 Department of Oncology, Lady Davis Institute for Medical Research of the Jewish General Hospital and McGill University, Montreal, Quebec, Canada

## 

It has long been recognized that dietary restriction (DR) can inhibit carcinogenesis. Recent work has not only clarified the mechanisms involved, which involve the effects of DR on circulating hormones and cytokines, but also demonstrated molecular characteristics of tumors that determine the extent to which they are influenced by variation in host energy intake. Importantly, we and others have also demonstrated that a subset of tumors are growth-stimulated by excess caloric intake, and provided strong circumstantial evidence that hyperinsulinemia is one of the mediating factors. Metformin, a biguanide used in cancer treatment, has been associated with reduced cancer risk in some hypothesis-generating pharmaco-epidemiologic studies. Laboratory studies have provided evidence for some antineoplastic activity. However, this activity is confined to subpopulations including subjects who are obese and/or hyperinsulinemic. Laboratory models suggest that metformin may act in this context because it is well-known to reduce hyperinsulinemia as a consequence of its reduction of gluconeogenesis and hyperglycemia. At the cellular level, metformin acts to inhibit oxidative phosphorylation, and this may provide an additional mechanism that could operate in the subset of cancers or at-risk tissues that express the cell surface transport molecules required for metformin entry to cells. The ATP deficiency induced by metformin may have little effect on some cells, an AMPK-dependent cytostatic effect on others, and still others may suffer an energetic crisis and necrotic death. Current research is providing additional details and mechanistic details underlying these actions, defining the subsets patients for which biguanides may be useful in cancer prevention or treatment, and defining relevant doses for potential new indications. Investigating the possibility of ‘repurposing’ metformin for applications in oncology is ongoing as laboratory and translational research ield data that will help to optimize clinical trial design.

